# The GWAS-MAP platform for aggregation of results of genome-wide association studies and the GWAS-MAP|homo database of 70 billion genetic associations of human traits

**DOI:** 10.18699/VJ20.686

**Published:** 2020-12

**Authors:** T.I. Shashkova, D.D. Gorev, E.D. Pakhomov, A.S. Shadrina, S.Zh. Sharapov, Y.A. Tsepilov, L.C. Karssen, Y.S. Aulchenko

**Affiliations:** Laboratory of Theoretical and Applied Functional Genomics, Novosibirsk State University, Novosibirsk, Russia; Laboratory of Theoretical and Applied Functional Genomics, Novosibirsk State University, Novosibirsk, Russia; Laboratory of Theoretical and Applied Functional Genomics, Novosibirsk State University, Novosibirsk, Russia PolyKnomics BV, ‘s-Hertogenbosch, The Netherlands; Laboratory of Theoretical and Applied Functional Genomics, Novosibirsk State University, Novosibirsk, Russia; Laboratory of Theoretical and Applied Functional Genomics, Novosibirsk State University, Novosibirsk, Russia; Laboratory of Theoretical and Applied Functional Genomics, Novosibirsk State University, Novosibirsk, Russia; PolyKnomics BV, ‘s-Hertogenbosch, The Netherlands; Laboratory of Theoretical and Applied Functional Genomics, Novosibirsk State University, Novosibirsk, Russia

**Keywords:** database, genome-wide association studies, quantitative genetics, varicose veins, GWAS-MAP, база данных, полногеномное исследование ассоциаций, количественная генетика, варикозная болезнь нижних конечностей, GWAS-MAP

## Abstract

Hundreds of genome-wide association studies (GWAS) of human traits are performed each year. The results of GWAS are often published in the form of summary statistics. Information from summary statistics can be used for multiple purposes – from fundamental research in biology and genetics to the search for potential biomarkers and therapeutic targets. While the amount of GWAS summary statistics collected by the scientific community is rapidly increasing, the use of this data is limited by the lack of generally accepted standards. In particular, the researchers who would like to use GWAS summary statistics in their studies have to become aware that the data are scattered across multiple websites, are presented in a variety of formats, and, often, were not quality controlled. Moreover, each available summary statistics analysis tools will ask for data to be presented in their own internal format. To address these issues, we developed GWAS-MAP, a high-throughput platform for aggregating, storing, analyzing, visualizing and providing access to a database of big data that result from region- and genome-wide association studies. The database currently contains information on more than 70 billion associations between genetic variants and human diseases, quantitative traits, and “omics” traits. The GWAS-MAP platform and database can be used for studying the etiology of human diseases, building predictive risk models and finding potential biomarkers and therapeutic interventions. In order to demonstrate a typical application of the platform as an approach for extracting new biological knowledge and establishing mechanistic hypotheses, we analyzed varicose veins, a disease affecting on average every third adult in Russia. The results of analysis confirmed known epidemiologic associations for this disease and led us to propose a hypothesis that increased levels of MICB and CD209 proteins in human plasma may increase susceptibility to varicose veins.

## Abstract (ru)

Ежегодно проводятся сотни полногеномных исследований ассоциаций (genome-wide association studies, GWAS) человеческих признаков. Результаты GWAS часто публикуют в форме сводных статистик. Информацию из сводных статистик можно использовать для решения различных задач – от фундаментальных исследований в области биологии и генетики до поиска потенциальных биомаркеров и мишеней терапевтического воздействия. Количество собранных научным сообществом сводных статистик GWAS быстро растет, однако использование данных затруднено из-за отсутствия общепринятых стандартов. В частности, исследователи, которые хотели бы применить сводные статистики GWAS в своей работе, сталкиваются с тем, что данные разбросаны по нескольким веб-сайтам, представлены в различных форматах, нередко без контроля качества. Более того, каждый доступный инструмент анализа сводных статистик запрашивает данные в своем собственном внутреннем формате. Для решения этих проблем мы разработали высокопроизводительную платформу GWAS-MAP для агрегации, хранения, анализа, визуализации и доступа к базе данных результатов полногеномных и региональных исследований ассоциаций. В настоящий момент на платформе содержится информация о более чем 70 миллиардах ассоциаций между вариантами геномной последовательности и болезнями, количественными и «омиксными» признаками человека. Платформа и база данных могут использоваться для изучения этиологии заболеваний человека, разработки предиктивных моделей риска, а также для поиска потенциальных биомаркеров и терапевтических воздействий. Применение платформы как инструмента для извлечения новых биологических знаний и формулировки гипотез о механизмах генетического контроля продемонстрировано на примере варикозной болезни нижних конечностей, заболевания, встречающегося у каждого третьего взрослого жителя России. Результаты проведенного анализа подтвердили известные эпидемиологические ассоциации для данного заболевания и позволили выдвинуть гипотезу о том, что уровень белков MICB и CD209 в плазме крови человека может влиять на риск варикозного расширения вен.

## Introduction

Genome-wide association studies (GWAS) are one of the main approaches for identifying associations between genetic variants and traits (Visscher et al., 2017). One of the most important advantages of this approach is that it is agnostic to the molecular mechanisms or biochemical nature of the traits or diseases under study, thus allowing fundamentally new knowledge to be obtained. Based on the functions of the genes mapped by a GWAS, researchers aim to discover new molecular mechanisms underlying the development of traits and pathologies under consideration.

GWAS are performed on large samples of genotyped and phenotyped individuals to identify statistically significant associations between single-nucleotide polymorphisms(SNPs) and traits (Bush, Moore, 2012). SNPs are located relatively homogeneously and with sufficient density, consequently, functional variants occurring at high frequency in the population are detected with a high probability, either because the causative allele is being tested directly or because it is in linkage disequilibrium with genotyped markers. A special case of GWAS is a regional genetic study of associations or a region-wide association study (RWAS), where the analysis is applied to SNPs in a particular region instead of the whole genome. RWAS is used, for example, to find cis-SNPs associated with the expression of a certain gene (GTEx Consortium et al., 2017).

The GWAS approach has become very popular over the past decade. Since 2007 the number of GWAS has increased exponentially and hundreds of original genome-wide studies are published every year. The earliest GWAS addressed the associations between a single trait and several hundreds of thousands SNPs, using samples of several hundreds or thousands individuals ( Klein, 2005; International Schizophrenia Consortium et al., 2009).

Currently, both the number of analyzed traits and the genomic coverage of GWAS have increased by many orders of magnitude (Timmers et al., 2019). This has become possible due to the advent of new sequencing and genotyping technologies and the improvement of existing ones, as well as other methods for studying biological objects, leading to an increase in the resolution of sequencing, genotyping and phenotyping. Modern GWAS normally assess associations with millions of SNPs and in some cases the sample size exceeds one million people (Timmers et al., 2019). The number of phenotypes studied can go to hundreds (Demirkan et al., 2012; Shen et al., 2017), thousands (Sun et al., 2018) and even tens of thousands (GTEx Consortium et al., 2017), e. g. for “-omics” traits. The same trait is analyzed in multiple studies, often with progressively increasing sample sizes, as well as in new populations, offering increased power and generalizability

The direct results of GWAS/RWAS consist of files with summary statistics. These files can include up to ten of millions rows, where each row contains information about the association between a given SNP and the investigated trait. Taking into account the number of GWAS studies and the size of the files with results, GWAS results qualify as Big Data (Wu et al., 2013; Fabregat-Traver et al., 2014). Importantly, not only does this body of data grow, but so do the rates of data acquisition. 

GWAS results can be used to address a large number of problems ranging from fundamental biology and genetics to the search for biomarkers and targets for therapeutic interventions. Currently, a range of methods has been developed that implement the solution of these problems based on summary statistics data.

In particular, methods have been developed to define sets of SNPs that are most likely to contain the true functional variant at loci suggested by GWAS (Kichaev et al., 2014; Benner et al., 2016; Schaid et al., 2018). For example, this problem is addressed by the PAINOR (Kichaev et al., 2014) software and the conditional and joint analysis as implemented in the GCTA tool (Yang et al., 2011).

Also, identification of causal genes influencing a trait of interest is possible through the use of summary statistics(Giambartolomei et al., 2014; Zhu et al., 2016; Momozawa et al., 2018). By regulating the expression of those genes or by manipulating their products through the use of, for example, pharmacological interventions, the trait of interest can be addressed in a targeted manner. Several instruments implement these methods, for example, the SMR (Summary-level Mendelian Randomization) tool (Zhu et al., 2016). The same methods can often be used to study pleiotropic effects (Klarić et al., 2020; Shadrina et al., 2020). The results of studies of pleiotropy can be used for drug repositioning, for predicting possible side effects of gene editing, and for prediction of possible side effects of pharmacological manipulation of the products of these genes

Over the past decade, the number of studies using Mendelian randomization methods has increased substantially, providing important new information about disease etiology (Elgaeva et al., 2019). Mendelian randomization methods combined with the use of summary statistics and multiple instrumental variables (Hemani et al., 2016; O’Connor, Price, 2018) can help to reconstruct the theoretical hierarchy of cause and effect relationships between traits and, in practice, have the potential to be used for the identification of traits that can be targeted by therapeutic interventions.

Methods for studying genetic correlations (Bulik-Sullivan et al., 2015; Speed, Balding, 2019) can be particularly useful in addressing fundamental questions related to the genetic architecture of complex traits. One of these methods is implemented in a popular LDsr (Linkage Disequilibrium score regression) python package (Bulik-Sullivan et al., 2015).

Finally, summary statistics from GWAS can be used in methods to develop models for the prediction of quantitative traits and disease risks for a given individual or group (Mak et al., 2017; Choi, O’Reilly, 2019; Lloyd-Jones et al., 2019). The simplest of these models use effects of the most significant independent SNPs (Evans et al., 2009). If a GWAS involves a large number of cases and controls, powerful predictors can be developed for some traits even with simple models, breast cancer being a well-known example (Mavaddat et al., 2019). Methods allowing the researcher to manage information about millions of SNPs and whole-genome LD structure while developing a prediction model have recently become popular (Vilhjálmsson et al., 2015). Such models were used for predicting the risk of ischemic heart disease (Khera et al., 2018), type 2 diabetes (Khera et al., 2018), and obesity (Khera et al., 2019).

Although the amount of GWAS results obtained by the scientific community is constantly growing, as are the number of methods for their analysis, they have currently found only limited use. The problems researchers face when working with these data are multiple. First, summary statistics files from GWAS are large (more than tens of terabytes), and so their storage and processing require dedicated infrastructure. Secondly, data are produced by different laboratories using different protocols, and consequently, quality control and a harmonization procedure for storing such data in a common format are required. Thirdly, the existing tools for analyses of summary statistics data from GWAS are implemented using different languages, hosted at different repositories and websites and require custom input data formats. Finally, large-scale adoption of these methods and data require user interfaces for researchers without specialized bioinformatics skills.

The existing solutions are incomplete or partial. On the one hand, resources such as GWAS Central (https://www. gwascentral.org) or GWAS Catalog (https://www.ebi.ac.uk/ gwas/) can do as much (that is, aggregate, store and provide access to GWAS results), but originally they were intended for handling “small data” (that is, the most statistically significant associations), and so their architecture does not scale well enough to handle big data and the requirements of new methods for processing GWAS results. On the other hand, most software applications (for example, SMR (Zhu et al., 2016), GCTA (Yang et al., 2011), LDsr (Bulik-Sullivan et al., 2015)) are intended for analyzing data rather than for aggregating, storing or providing access to them. Finally, the portals MR-Base (http://www.mrbase.org/) and LD Hub (http://ldsc.broadinstitute.org/) can aggregate and store GWAS results and allow the user to conduct specify types of analysis; however, these portals do not offer anything for other methods of analysis, and software solutions for data aggregation and storage are not available.

To help address these issues, we developed the GWASMAP platform for aggregating, storing, analyzing, visualizing and providing access to big data obtained from GWAS. The name GWAS-MAP means both a map between phenotypes and genotypes, but is also an abbreviation of Multiple Analyses Platform. Using GWAS-MAP we collected GWASMAP|homo database of GWAS and RWAS results for human traits. Currently, the database contains more than 70 billion associations between SNPs and human traits. GWAS-MAP provides an opportunity to carry out research that will contribute to the search for new biomarkers that has a bearing on the development of high-efficacy drugs and also reveal side effects in existing drugs. We have performed a genetic analysis of varicose veins to demonstrate how the platform works.

## The GWAS-MAP platform

GWAS-MAP software architecture

The GWAS-MAP platform consists of two data processing modules (one for integration and one for analysis of GWAS/ RWAS results) and a database (DB) module (see the Figure).

**Fig. 1.**
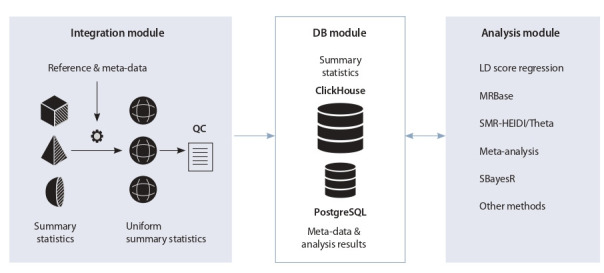
GWAS-MAP software architecture. Grey blocks: data processing modules. White block: database module with database management systems. Arrows between modules: saving/retrieving data to/from the DB. QC, quality control; DB, database; LD, linkage disequilibrium. Description of the software modules is provided in the text.

Data integration starts with the conversion of summary statistics files collected from various sources into a universal data format. After conversion, we perform quality control (QC) and if the summary statistics pass, they are uploaded to the databases.

The DB module is the part responsible for setting up the databases and tables structure required for the GWAS-MAP platform. The DB module consists of two components, each controlled by a separate open source database management system (DBMS). One of the components is used to store the GWAS summary statistics; for this component the ClickHouse DBMS version 19.16.2. revision 54427 (https://clickhouse. tech/) is used. Arecord in this system contains the parameters of association between certain SNP and a trait. The other component contains (1) meta-data that gives particular information about the summary statistics collected from articles, study web-sites or other sources, and (2) the results of analyses; for this component the PostgreSQL DBMS version 10.6 (https:// www.postgresql.org/) is used.

With the analysis module a user can run various analyses on the GWAS/RWAS summary statistics using the integrated analytical tools written in Python, which are accessible through command-line utilities.

Integration and quality control of GWAS/RWAS results

The platform offers users the option to upload GWAS summary statistics files of their own original research. Because these data were generated using different protocols, the resulting summary statistics files may appear in different formats. To address this, GWAS-MAP provides an integration module converting summary statistics files to a common format and performing QC on the data.

To ensure data consistency within the DB, information about a SNP’s identifier, position in the genome, and alleles and allele frequencies are compared with reference data. The reference is a list of SNPs with their main characteristics: the identifier (rsID), chromosome, position, alleles and allele frequencies. At present, the reference is based on the 503 genomes of European-ancestry individuals from the “1000 Genomes” project phase 3 version 5 (The 1000 Genomes Project Consortium et al., 2015).

In general, summary statistics contain all fields required for unification in a universal format. If some fields are absent, then the missing information is added from the reference (for example, allele frequency) or calculated from the information in the input file. For example, it is possible to recover the standard error of the effect size based on the effect size and p-value.

Before uploading GWAS/RWAS data to the DB, it is absolutely necessary to have them passed through QC. QC is indispensable not only for meta-analyses of GWAS/RWAS results, but also for verifying separate studies, because seemingly insignificant data errors may lead to heavily biased results later on.

We have developed a QC module which spots outlying SNPs (that is, those with characteristics other than expected) and assesses the overall quality of the input data. More specifically, QC includes (1) a comparison of the frequencies of alleles from the input data with those from the reference set, a comparison of the p-values provided in the study and those calculated from the Z-statistics (if present), (2) an analysis of the distribution of estimates of the allele effect sizes, (3) calculation of the trait variance and (4) genomic control factor (λGC). SNPs whose characteristics depart by more than a threshold value from those expected are labeled as outliers and can be filtered out by the user. If the summary statistics from GWAS have more than 5 % outliers, or the effect size distribution is not symmetric, this data will be not recommended for upload, although the final decision is up to a user. We should notice that all current data in DB have passed the above described criteria.

Analysis methods using GWAS/RWAS summary statistics implemented in GWAS-MAP

GWAS-MAP incorporates several widely used methods for the analysis of GWAS/RWAS summary statistics with special emphasis on the identification of genes, molecules, traits and functional SNPs that appear as potential targets of therapeutic interventions. In particular, data processing can be carried out using the following methods.

- Linkage disequilibrium score regression is a method to assess the heritability of a trait and to calculate genetic correlations between two traits (Bulik-Sullivan et al., 2015). This method was implemented in Python 2 by Bulik-Sullivan and co-authors (2015). We have re-written it in Python 3 because it is the main programming language used for GWAS-MAP and because Python 2 has been deprecated since January 1, 2020. This also allowed us to optimize it for working with our DBs.
- Mendelian randomization methods – a set of tests that allow to infer causal relationships between two traits (Hemani et al., 2016). Hemani and colleagues provided an open source R package, which includes such methods. To this, we added a module for reading summary statistics from the GWAS-MAP DB in the required format
- Summary-level Mendelian randomization (SMR) and heterogeneity in dependent instruments (HEIDI) are the tests to ascertain whether two different traits are associated with the same locus (SMR) and whether this association can be explained by the null hypothesis of pleiotropy or by an alternative hypothesis that each trait is associated with different SNPs in linkage disequilibrium (LD) (HEIDI) (Zhu et al., 2016). We implemented the SMR-HEIDI tests ourselves for the GWAS-MAP platform. The rationale behind this was mainly that the SMR tool developed by Zhu and colleagues (2016) specializes in testing pleiotropy between the level of gene expression (RWAS) and a complex trait (GWAS), but not between two sets of GWAS results summary statistics.
- We also implemented the θ metric defined by Momozawa et al., which assesses the similarity between association profiles using only summary statistics and is an alternative to the HEIDI test. This method is preferable when the LD information of the population used in a GWAS is lacking or unreliable (Momozawa et al., 2018). The θ metric as implemented in GWAS-MAP is based on the equations provided in the article (Momozawa et al., 2018).
- Finally, the GWAS-MAP platform implements several standard methods for meta-analysis which can be applied to a pool of GWAS results of the same trait in order to obtain enhance power (Winkler et al., 2014). GWAS-MAP has a module for checking the quality of the GWAS results to be used in meta-analyses and a module for meta-analysis. We have implemented two methods for meta-analysis: inverse-variance weighting and Z-score (Evangelou, Ioannidis, 2013).

GWAS-MAP|homo database content

To allow the researcher to filter GWAS/RWAS according to certain criteria, the platform offers key information including the publication data, reference set used for imputations, the name/type of the DNA microarray (e.g. Metabochip, Affymetrix, Illumina SNP arrays) or whole-genome sequencing used in each study. Currently, the DB contains more than 70 billion associations between SNPs and traits, collected from 7281 GWAS and more than a million RWAS (see the Table). To give a reader an idea of the context, such popular databases as “GWAS central” (Beck et al., 2020) provides information on 71 million of associations, while Phenoscanner (Staley et al., 2016) – 65 billion.

**Table 1.**
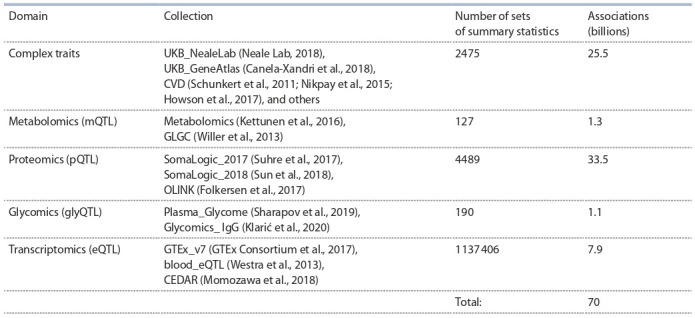
Database content Note. List of collections in the DB, the domains to which they have been assigned and the corresponding numbers of GWAS/RWAS and SNP summary statistics. Domains: complex traits, mQTL (metabolite levels), pQTL (protein levels), glycomics (glycan levels), and eQTL (gene expression data). UKB, UK Biobank; CVD, cardiovascular diseases; GLGC, Global Lipids Genetics Consortium; IgG, immunoglobulins G.

The GWAS and RWAS in the DBs are assigned to the following domains: complex traits (including diseases), metabolites (mQTL), proteins (pQTL), glycans and gene expression data. Additionally, the GWAS and RWAS results coming from the same study are pooled in a collection. The presence of GWAS traits from different domains enables the researcher to conduct a comprehensive study of the trait of interest, to identify ways of how the trait of interest is influenced by the expression levels of genes, proteins and metabolites, and to look for associations with other diseases or quantitative traits.

## GWAS-MAP application: a genetic analysis of varicose veins

Varicose veins (VV) is a widely prevalent disease affecting on average every third adult in Russia (Zolotukhin et al., 2017). The genetic basis of this pathology has long been poorly studied. Shadrina and co-authors have performed the first large-scale study of its genetic architecture using a range of modern methods in bioinformatics as implemented in GWASMAP (Shadrina et al., 2019)

The study used UK Biobank (http://www.ukbiobank.ac.uk/) data on 408,455 individuals of European descent. GWAS summary statistics of VV were retrieved from the open access databases Gene ATLAS (Canela-Xandri et al., 2018) and the Neale Lab website (Neale Lab, 2018). Shadrina and co-authors identified 12 genetic loci associated with VV which account for 13.4 % of the SNP-based heritability. A gene or a group of genes most probably involved in VV pathogenesis was prioritized for each locus. The SMR-HEIDI implementation in GWAS-MAP was used as one of the prioritization methods. With SMR-HEIDI, we searched for the genes for which the expression levels are associated with SNPs affecting VV risk (cases of the so-called colocalization of associated loci). The analysis relied on data from the eQTL (expression quantitative trait loci) domain, namely data of 44 tissues in the GTEx_v7 (GTEx Consortium et al., 2017) and blood_eQTL (Westra et al., 2013) collections. Colocalization was demonstrated for the following loci: rs3101725 (associated with the expression level of the long non-coding RNA LINC01184 in 9 tissues), rs2241173 (associated with the expression level of the non-coding RNA AC005152.3 in the lower extremity skin) and rs2861819 (associated with expression levels of the PPP3R1 gene in blood). Because the functions of LINC01184 and AC005152.3 are not yet known, we may only speculate about the role of these RNAs in VV. As far as PPP3R1 is concerned, its association with VV appears to be more sound. Its product is involved in the inflammatory response in the vascular wall, stimulating the production of the chemokine MCP-1 (Satonaka et al., 2004), which is consistent with the modern view of the pathogenesis of chronic venous disease (Lim, Davies, 2009; del Rio Solá et al., 2009). Additionally, Smetanina and co-workers demonstrated enhanced PPP3R1 expression in VV specimens compared to unaffected veins (Smetanina et al., 2018).

In addition to gene prioritization, SMR-HEIDI was used to search for traits associated with VV-related functional variants. The analysis involved 2219 traits, including various diseases, levels of metabolites and proteins in blood, and revealed 32 traits associated with 6 loci. The traits can conventionally be divided into three main groups: one associated with body weight and the total metabolic rate; a second with blood test results, and a last one with all others.

The GWAS-MAP platform was also used for the analysis of genetic correlations between VV and 861 traits, the summary statistics of which were obtained by analyzing more than 10 thousand individuals. The analysis showed the presence of common genetic variance between VV cases and 62 traits. Some of these traits were already known from previous epidemiological studies: overweight, standing and heavy physical work, deep venous thrombosis, gonarthrosis, and pain in the legs when walking. Other traits that, at the genetic level, correlate with VV, such as intellect, memory, educational attainment, or whole-body pain, have not previously been reported as associated with VV

Finally, Shadrina and co-workers used Mendelian randomization for the analysis of causal relationships between various traits and VV. Analysis results showed that the following traits directly influence the risk for VV: height (irrespective of weight), body weight; waist and hip circumferences, and the blood levels of two proteins, MICB and CD209 (also known as DC-SIGN). Curiously, the risk for VV increased with an increase of both body fat and fat-free mass. Data on height as a risk factor for VV are consistent with the Edinburgh Vein Study results (Lee et al., 2003). MICB and CD209 participate in the innate and the adaptive immune response. Because presented work is the first to propose that these proteins have roles in VV pathogenesis, we think it reasonable to repeat the analysis with an independent dataset. If the Mendelian randomization results are confirmed, these proteins can be regarded as promising candidates for further in vivo and in vitro studies aimed at finding therapeutic targets.

## GWAS-MAP benefits and future development

The GWAS-MAP platform offers a broad range of opportunities for comprehensive analysis of GWAS results. We expect that GWAS-MAP will be helpful both for bioinformatics studies and as a reference source for medical researchers. For example, given a trait of interest, it is possible to compute what other traits it is genetically associated with, i. e., is controlled by overlapping sets of genetic variants. More generally, not only correlations between traits can be calculated, but also all pairwise correlations between the traits in the DB. The results can be used to cluster the traits and/or to build a network connecting traits that have a shared genetic basis (see, for example, Fig. 4 from Shadrina et al., 2019). Furthermore, the Mendelian randomization methods implemented in the platform will help to elucidate which of these associations are causal. Thus, it is possible to build a directed graph for interactions between traits. By considering a particular vertex, for example, “disease”, it is possible to infer what metabolites, glycans and/or proteins can be used as its biomarkers.

If a researcher’s interest lies with a locus or loci associated with a certain GWAS of interest, it is also interesting to consider colocalization. With SMR-HEIDI and the θ metric, it is possible to understand with the expression of what genes the GWAS loci are associated. Additionally, by analysis of RWAS results for the genes of interest and GWAS results in the domains for metabolites, proteins and/or glycans, it is possible to infer what biological processes are associated with changes in the expression of these genes. A large-scale analysis of colocalization will help to build networks of associations between traits in the DB and genes. These networks will be helpful in developing medications. Not only will they show what genes can be targeted, but also what implications and side effects of manipulations with the gene may entail. However, it should be kept in mind that these analyses are done in silico and therefore, and experimental validation is absolutely required.

A large number of methods have already been implemented in the platform – however, there are certainly more to come. Our short-term plans include the addition of new analysis methods: Depict (Pers et al., 2015), CoJo (Deng, Pan, 2018), and SbayesR (Lloyd-Jones et al., 2019). We are planning to develop a web-interface to allow external users to access our DBs and perform analyses. Such a web-interface will guide the user through the search for information about the association between a SNP and traits and will be convenient for e. g. medical researchers. We continue adding new data to the database and we are working on making GWAS-MAP useful for human populations other than Europeans.

## Conclusion

We have developed the GWAS-MAP platform for aggregating, storing, analyzing, visualizing and providing access to summary statistics from GWAS and RWAS. Using the platform we collected GWAS-MAP|homo DB which contains over 70 billion associations between SNPs and traits. The GWAS-MAP user interface offers a universal workspace for operating on public and private data, and allows for rapid implementation of new analysis methods in the platform. The user communicates with the platform through command-line utilities, allowing him to upload data to the platform and run analyses.

The analysis of the genetic basis of varicose veins demonstrates the power of the platform for generating new biological hypotheses such as, for example, ours postulating a causal relationship between the levels of the proteins MICB and CD209 in blood and the risk for this disease.

GWAS-MAP is a powerful platform for the analysis of summary statistics from GWAS and RWAS, it is actively used in research work and can be useful to a broad range of scientists. The platform evolves continuously through constant acquisition of more functionalities, and the DBs are updated with the actual data from GWAS and RWAS.

## Conflict of interest

The authors declare no conflict of interest.
